# *Trypanosoma cruzi* (DTU TcI) in a fatal case of meningoencephalitis due to Chagas disease reactivation in a patient coinfected with human immunodeficiency virus: case report

**DOI:** 10.1590/0037-8682-0234-2024

**Published:** 2025-10-03

**Authors:** Hevillyn Fernanda Lucas da Silva, Luís Fernando Fernandes Miranda, Matheus da Silva de Oliveira, Pedro Paulo Gilio Saraiva, Rafael Zanutto Nakata, Rodrigo Pernomian Cianca, Rúbia Pazzetto, Cesar Helbel, Amanda Regina Nichi de Sá, Cristiane Maria Colli, Max Jean de Ornelas Toledo

**Affiliations:** 1 Universidade Estadual de Maringá, Programa de Pós-Graduação em Ciências da Saúde, Maringá, PR, Brasil.; 2 Universidade Estadual de Maringá, Departamento de Ciências Básicas da Saúde, Maringá, PR, Brasil.; 3 Universidade Estadual de Maringá, Hospital Universitário Regional de Maringá, Maringá, PR, Brasil.; 4 Universidade Estadual de Maringá, Departamento de Medicina, Maringá, PR, Brasil.; 5 Universidade Federal da Grande Dourados, Faculdade de Ciências da Saúde, Dourados, MS, Brasil.

**Keywords:** DTU TcI, Chagas disease, Human immunodeficiency virus, Central nervous system, Meningoencephalitis

## Abstract

The reactivation of Chagas disease (RCD) by human immunodeficiency virus (HIV) is associated with high mortality and is a relevant public health problem in areas endemic for the causative agent of CD, *Trypanosoma cruzi* (*T. cruzi*). Here, we report a fatal case of meningoencephalitis caused by RCD involving the discrete typing unit (DTU) of *T. cruzi* I (TcI) in an HIV-coinfected patient from Paraná, Brazil. Based on computed tomography findings, a 55-year-old man initially underwent empirical treatment for neurotoxoplasmosis. However, Giemsa-stained cerebrospinal fluid and blood smears revealed *T. cruzi* trypomastigotes on direct microscopic examination. Protozoa were detected by fresh blood examination and blood culture. Additionally, anti-*T. cruzi* immunoglobulin G antibodies were detected in serum using a chemiluminescent immunoassay. Blood culture sequencing of cytochrome oxidase II confirmed the DTU TcI infection. Benznidazole therapy was administered for 76 d; however, the patient showed no clinical improvement and died nearly 7 months after hospital admission. The fatal outcome was likely related to delayed diagnosis and treatment, severe immunosuppression (CD4 = 39 cells/mm³), high viral load (94,638 copies/mL), and the involvement of TcI, which has been consistently associated with fatal RCD-related meningoencephalitis.

## INTRODUCTION

Reactivation of Chagas disease (RCD) has been established as an acquired immunodeficiency syndrome (AIDS)-defining condition^1^ ^,^ ^2^, and human immunodeficiency virus (HIV)-*Trypanosoma cruzi* coinfection is associated with high mortality^1^ ^,^ ^2^.

RCD can manifest as myocarditis or meningoencephalitis^2^ ^,^ ^3^. RCD involving the central nervous system (CNS) can be fatal. Therefore, early detection and treatment are necessary to maximize survival^3^. Etiological confirmation of RCD, particularly with CNS involvement, should follow a hierarchical approach based on diagnostic specificity. The gold standard is the detection of *T. cruzi* amastigotes in brain tissue obtained through biopsy, surgical procedures, or autopsy. Direct microscopic observation of motile trypomastigotes in fresh cerebrospinal fluid (CSF) preparations provides rapid evidence of high parasitemia in the CNS. In cases where microscopy is negative, but clinical and radiological suspicion remains high, quantitative real-time polymerase chain reaction (qPCR) for *T. cruzi* in the CSF can quantify parasitemia and identify a patient coinfected with HIV/*T. cruzi* infection with reactivation. An increase in HIV viral load, a decrease in the number of CD4+ cells/mm3, and the CD4+/CD8+ ratio are cofactors for increased parasitemia that can be used to target the introduction of early, pre-emptive therapy^4^. Neuroimaging, such as computed tomography or magnetic resonance imaging, may reveal mass-effect lesions suggestive of Chagasic meningoencephalitis, although these findings are not pathognomonic. In addition, when cardiac reactivation is suspected, electrocardiography and echocardiography may detect signs of acute myocarditis, contributing to clinical assessment even though they do not confirm the etiology on their own^2^, and ruling out other diagnostic hypotheses^3^.

Although serological testing for *T. cruzi* is essential to establish prior exposure and to confirm chronic Chagas disease (CD), it does not distinguish between latent infections and reactivation. Furthermore, in immunosuppressed individuals, such as patients with HIV/AIDS, antibody production may be impaired, potentially leading to false-negative results. In contrast, molecular methods, such as qPCR, are essential for confirming reactivation, as they enable direct detection and quantification of circulating *T. cruzi* DNA in the blood or CSF. This technique is highly sensitive, particularly during reactivation when parasitemia is elevated, and can provide positive results even when microscopy is negative. Thus, qPCR is a valuable tool for prospective monitoring of *T. cruzi* parasitemia in patients coinfected with HIV and etiological confirmation of RCD, and may also contribute to monitoring treatment response^2^ ^,^ ^4^ ^,^ ^5^.

Currently, seven *T. cruzi* discrete typing units (DTUs) have been recognized: TcI-TcVI and Tcbat^6^ ^,^ ^7^. This genetic diversity can significantly influence the clinical presentation, prognosis, and treatment response during RCD^6^ ^,^ ^7^. Evidence suggests that certain DTUs such as TcI and TcII are associated with distinct clinical manifestations and tissue tropism patterns that may affect the severity of reactivation, particularly in immunosuppressed patients. Moreover, genetic variations among parasitic strains may affect susceptibility to benznidazole (BZ) and other trypanocidal drugs, thereby influencing therapeutic efficacy^8^. Thus, identifying the DTU (s) involved may provide prognostic information and support individualized clinical decision-making. However, information on the DTUs involved in RCD in patients with HIV/AIDS, including the frequency of this association, survival/death rates, recommendations for an optimal treatment regimen, and clinical outcomes based on DTU, is scarce.

We report a fatal case of RCD meningoencephalitis in a patient coinfected with HIV and *T. cruzi* (DTU TcI) and review the literature on this topic. Using the terms “HIV or reactivation,” “Chagas disease or *T. cruzi*,” and “DTU or lineages or genotype” in the Cochrane Library, LILACS, SciELO, MEDLINE/PubMed, PubMed Central, EMBASE, Web of Science, Scopus, and Portal Capes databases, including secondary references, we identified eleven relevant publications (Table 1) on *T. cruzi*-HIV coinfection^9^ ^-^ ^19^, except publications of Cura et al. (2012)^14^ and (2015)^17^ in which 34 patients presented with clinical reactivation in the context of immunosuppression (33 transplant recipients and one with lymphoma). We aimed to identify the DTUs present in cases of reactivation involving the CNS, and to characterize the general aspects observed in these cases. All 11 publications were considered in the analyses, including 5 published before the division of *T. cruzi* into DTUs^9^ ^-^ ^13^. In earlier studies, DTUs were inferred based on previously used classifications^20^. 

**TABLE 1:** Clinical presentation, underlying disease, geographic origin, and *Trypanosoma cruz*i discrete typing units (DTUs) identified in patients with Chagas disease and immunosuppression, with or without clinical reactivation.

Sample size, clinical presentation and underlying disease	Geographic origin of patients	Discrete typing unit (DTU) *	Reference (authors and year) ^#^
1 patient with central nervous system (CNS) reactivation due to *T. cruzi*/HIV coinfection	Brazil, Minas Gerais	TcVI (Genotypically like the CL strain and zymodeme 2)	Pacheco et al. (1998)^9^
1 patient with CNS reactivation due to *T. cruzi*/HIV coinfection	Brazil, Minas Gerais	TcII - TcVI (genetic group 2)	Lages-Silva et al. (2002)^10^
1 patient with CNS reactivation due to *T. cruzi*/HIV coinfection	Argentina	TcV (TcIId): Blood TcII (TcIIb) + TcVI (TcIIe): Brain	Burgos et al. (2005)^11^
1 patient with CNS reactivation due to *T. cruzi*/HIV coinfection	Bolivia	TcI: Blood; TcV/VI (TcIId/e): Blood; TcI: CSF	Burgos et al. (2008)^12^
19 patients: 7 mothers with the indeterminate CD (6^) and encephalitis (1); 2 infants with congenital CD^; 10 adults with CNS reactivation (7), indeterminate CD (2^) and unknown (1). All coinfected with *T. cruzi*/HIV	Argentina (10 patients), Bolivia (1) and unknown (8)	9 patients with TcV; 2 patients TcII/V/VI; 2 patients TcI + TcV; 1 patient: not determined	Bisio et al. (2009)^13^
23 patients/samples with reactivation in the context of immunosuppression due solid organ transplant or *T. cruzi*/HIV coinfection	Argentina	11 samples: TcI; 4 samples TcV; 4 samples TcV + TcII/VI; 2 samples: TcII + TcVI; 2 samples not detected	Cura et al. (2012)^14^
1 patient with CNS reactivation due to *T. cruzi*/HIV coinfection	Colombia	Heart with TcI + TcII and brain with TcI	Hernández et al. (2014)^15^
1 patient with CNS reactivation due to *T. cruzi*/HIV coinfection	Brazil, rural area of Bahia	TcII	Buccheri et al. (2015)^16^
12 patients with reactivation in the context of immunosuppression (11 transplanted and 1 with lymphoma)	Argentina	3 patients with TcI; 1 patient with TcII; 1 patient with TcV; 1 patient with TcII/VI	Cura et al. (2015)^17^
8 coinfected *T. cruzi*/HIV patients under antiretroviral therapy without reactivation	Brazil, São Paulo	2 patients with TcII and 1 patient with TcII + TcV/VI	Marcon et al. (2022)^18^
8 patients with CNS reactivation due to *T. cruzi*/HIV coinfection	Argentina (5) and Paraguay (3)	4 patients with TcV + TcVI: blood and CSF (2) and blood (2); 3 patients with TcV: blood and CSF (2) and blood (1); 1 patient with TcVI: blood and CSF	Fernández et al. (2023)^19^

* Zingales et al. (2009)^20^; ^#^ Publications found in the search using seven terms (“HIV or reactivation,” and “Chagas disease or *T. cruzi*,” and “DTU or lineages or genotype”) and 10 databases (Cochrane Library, LILACS, SciELO, MEDLINE/PubMed, PubMed Central, EMBASE, Web of Science, Scopus, Biblioteca Brasileira de Teses e Dissertações and Portal Capes); ^ Absence of reactivation of Chagas disease.

## CASE REPORT

**FIGURE 1:**
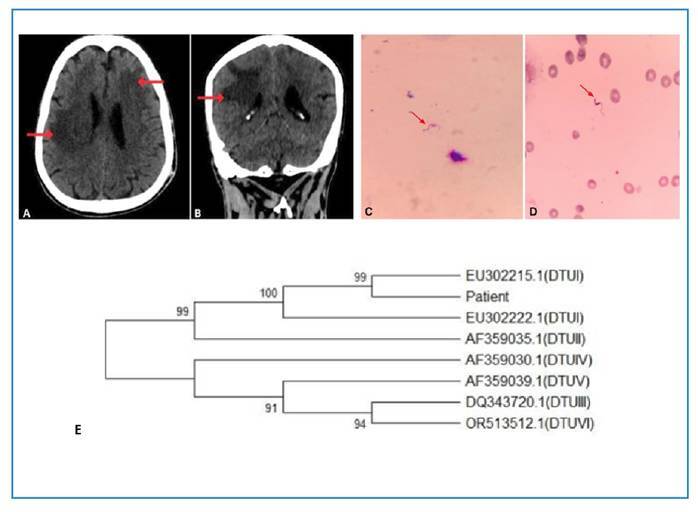
Computed tomography of the head: **(A)** in the cross-section, hypoattenuation in the right temporoparietal region and in the left frontal region, both with vasogenic edema; **(B)** in the coronal section, hypoattenuation in the right temporoparietal region. *Trypanosoma cruzi* trypomastigote form observed in a Giemsa-stained smear of cerebrospinal fluid **(C)** and blood **(D)** from the patient (magnification 1000×). Dendrogram depicting the phylogenetic relationships between the patient isolate and the reference strains of the six discrete typing units (DTUs) of *Trypanosoma cruzi*, based on sequences of the mitochondrial cytochrome oxidase subunit II (COII) gene. The analysis included COII sequences from known molecular patterns of *T. cruzi*, as Silvio (DTU I - GenBank EU302222.1), Esmeraldo (DTU II - GenBank AF359035.1), 231 (DTU III - GenBank DQ343720.1), CANIII (DTU IV - GenBank AF359030.1), SO3cl5 (DTU V - GenBank AF359039.1), and CL Brener (DTU VI - GenBank OR513512.1). GenBank accession EU302215.1 (DTUI) was included in the analysis because it presented the highest similarity with the sequence obtained from the patient sample, as identified by BLAST software search. The COII sequences of *T. cruzi* isolated from the patient were submitted to GenBank for analysis and deposit in the genetic archive. The phylogenetic analyses and the neighbour-joining tree were conducted using software MEGA version 12^23^ **(E)**. *Source:* the authors.

In November 2020, a 55-year-old retired man with a history of alcohol and tobacco use and no known family ties was admitted to the emergency room at Hospital Universitário Regional de Maringá in a disoriented state with left hemiparesis and dysarthria. He reported a childhood brain injury previously treated with an unspecified medication, which was discontinued owing to alcohol use and not resumed, despite 1 year of abstinence. On admission, the patient presented with malaise, tongue rigidity, and vomiting. He had not sought medical care for approximately 5 years. He was born in the municipality of Terra Boa, and lived in the municipality of Paiçandu, Northwest of Paraná, Brazil. Computed tomography (CT) of the head revealed hypoattenuation of the right frontal, parietal, and temporal lobes (Figure 1A and B ). The patient was started on acetylsalicylic acid and simvastatin owing to the suspicion of stroke and was subsequently moved to a ward.

Upon admission, a rapid HIV test yielded a positive result; however, this condition was unknown to the patient and his family. 

As the patient showed no clinical improvement by 17 d after admission (d.a.a.), CT was repeated on December 5, 2020, which revealed typical signs of neurotoxoplasmosis; thus, empiric treatment for this infection was started with 75 mg/day pyrimethamine, 1,500 mg sulfadiazine, and 4 mg dexamethasone every 6 h, and 15 mg/day folinic acid. Two additional CT scans were performed during hospitalization, during which the same radiological findings were observed.

Meningoencephalitis caused by RCD was diagnosed at 22 d.a.a. (December 10, 2020) following direct blood and CSF examination of material collected at 20 d.a.a., which had a slightly cloudy appearance, together with CT signs of meningoencephalitis that were thought to be neurotoxoplasmosis-related. CSF analysis revealed pleocytosis with a predominance of mononuclear leukocytes, glucose and protein levels within the reference values (Table 2), and the presence of flagellated protozoa. The CSF and whole blood samples were sent to the Chagas Disease and Molecular Parasitology Laboratory at the State University of Maringá (UEM) for parasitological analyses. After Giemsa staining of the CSF and blood smears, direct examination by optical microscopy led to the identification of trypomastigote forms of *T. cruzi* (Figure 1C and D ). The patient was subsequently started on etiological treatment with benznidazole (100 mg every 8 h). A fresh blood examination and blood culture (in liver infusion tryptose medium) were positive for *T. cruzi*. Furthermore, anti-*T. cruzi* IgG antibodies were detected in the serum using a chemiluminescent microparticle immunoassay carried out at the Teaching, Research, and Clinical Analysis Laboratory of UEM. 

**TABLE 2:** Laboratory results at the time of diagnosis of Chagas disease reactivation, during and after treatments.

Days after admission	0	17	20	22^a^	55^b^	66	73	100	132	138	Reference values
Blood count											
Red blood cells (millions/μL)	4.25	3.55	3.54	3.31	2.19	2.90	2.62	2.9	2.82	3.58	4.50 - 5.90
Hemoglobin (g/dL)	12.0	9.9	9.8	9.3	5.9	7.5	7.3	8.1	8.6	11.2	13.5 - 17.5
Hematocrit (%)	36.0	29.7	29.4	27.5	18.1	23.0	21.4	24.3	25.2	33.1	41.0 - 53.0
Total leukocytes (mm^3^)	4,990	5,400	14,120	9,600	5,270	11,030	7,743	6,205	5,069	6,160	4,000 - 10,000
Platelets (mm^3^)	186,000	216,000	257,000	160,000	274,000	295,000	220,600	169,900	185,500	273,700	150,000 - 450,000
*T. cruzi* on direct examination	-	-	Yes	-	-	-	-	-	-	-	-
CSF routine											
Aspect	-	Clear	Cloudy	Clear	-	-	Cloudy	-	-	-	Clear
Color	-	Colorless	Colorless	Colorless	-	-	Erythrochromic	-	-	-	Colorless
Global cytology											
Leukocytes (mm³)	-	6	19	24	-	-	2	-	-	-	0 - 5
Red blood cells (mm^3^)	-	186	933	3,856	-	-	5,120	-	-	-	0
Differential cytology											
Polymorphonuclear (%)	-	*	1	2	-	-	*	-	-	-	-
Mononuclear (%)	-	*	99	98	-	-	*	-	-	-	-
Eosinophils (%)	-	*	0	0	-	-	*	-	-	-	-
Biochemical analysis											
Glucose (mg/dL)	-	73.0	65.0	70.0	-	-	49.0	-	-	-	40.0 - 70.0
Proteins (mg/dL)	-	68.0	59.0	28.0	-		113.0	-	-	-	12.0 - 60.0
Lactate (mg/dL)	-	11.8	18.0	10.9	-	-	12.6	-	-	-	5.4 - 19.8
*T. cruzi* on direct examination	-	no	Yes	yes	-	-	no	-	-	-	-
Viral load and CD4 cell quantification											
CD4 count (cells/mm³)	-	-	-	-	39	-	-	-	99	-	> 350
HIV-1 viral load (copies/mL)	-	-	-	-	94,638	-	-	-	nd	-	-
Other exams											
Creatinine (mg/dL)	0.66	0.57	0.60	0.59	0.57	3.99	3.76	0.35	-	0.56	0.66 - 1.25
AST (U/L)	18	-	-	-	-	29	-	-	-	-	17 - 59
ALT (U/L)	11	-	-	-	-	22	-	-	-	-	< 50

*differential leukocyte counts not performed because of low leukocyte count; **a:** start of antitrypanosomal treatment; **b:** start of antiretroviral treatment; **-:** unperformed; **nd:** not detected; **AST:** aspartate aminotransferase; **ALT:** alanine aminotransferase.

In order to rule out other diagnostic hypotheses, tests for *Mycobacterium tuberculosis*, *Cryptococcus neoformans*, and cytomegalovirus yielded negative results. However, low concentrations of anti-*Toxoplasma gondii* antibodies were found in the CSF (0.20 and 0.03 IU/mL of immunoglobulin (Ig)G and IgM, respectively), and thus, empiric treatment for toxoplasmosis was maintained.

An HIV viral quantification test and CD4 + T lymphocyte count performed on January 12, 2021 (55 d.a.a.) revealed a high viral load and low lymphocyte count (Table 2). Antiretroviral treatment (ART) with 300 mg tenofovir, 50 mg dolutegravir, and 300 mg lamivudine was started on this date, in expectation of the patient's clinical improvement, since he was very weak upon admission. Weekly assessments, including ECG monitoring, were conducted to identify possible adverse reactions to medications, such as changes in liver and kidney function and/or cardiotoxicity.

Despite initiating RCD and HIV treatments, the clinical picture worsened, and the patient displayed a decerebrate posture, diffuse hypertonia, and deviation of gaze to the right, in addition to acquiring nosocomial infections that were treated with antibiotics. No typical CD changes were observed on transthoracic echocardiography or esophagogastroduodenoscopy. The patient completed 76 d of RCD treatment, was discharged to the care of his family on April 5, 2021 (138 d.a.a.) without clinical improvement and died 1 month later. According to the official death certificate (issued on May 7, 2021), no autopsies were performed. The documented causes of death were as follows: R98, unattended death; B58.2, toxoplasmic meningoencephalitis (listed as the underlying cause); B57.2, chronic Chagas disease with cardiac involvement; and I69.4, sequelae of an unspecified cerebrovascular accident.

Although *T. cruzi* was detected by direct microscopic examination of both the blood and CSF, indicating a high parasitic load, initial attempts to genotype the parasite using two different molecular methods were unsuccessful. PCR analyses using 24Sα ribosomal DNA (rDNA) and cytochrome oxidase II (COII) markers were performed directly on these biological samples, but did not result in amplification, indicating an insufficient amount of parasite DNA. Detection and molecular characterization were only possible using DNA extracted from positive blood cultures, highlighting the limitations of direct genotyping methods that rely on the amount of DNA present in the sample. We clarified that conventional PCR was employed in this study because qPCR methodology was not available in our laboratory at the time, making its use for patient sample analysis unfeasible.

PCR was performed to amplify the 24Sα rDNA sequence of *T. cruzi* isolated from the blood culture for genotyping^21^. After separation on a 6% polyacrylamide gel, bands equivalent to those of the reference strains, corresponding to TcI, TcIII, and TcV, were observed in the patient's blood culture, presenting two amplified products of approximately 110 and 125 bp. To determine the DTU involved, PCR of the COII gene was performed^21^ and the amplicons were sequenced in both directions according to the manufacturer’s instructions with the BigDye Terminator v3.1 Sequencing Kit (Thermo Fisher Scientific) in an automated DNA sequencer (3500xL, Applied Biosystems). Nucleotide sequences were analyzed and edited using bioinformatics tools such as reverse complementation (https://www.bioinformatics.org/sms/rev_comp.html) and Pairwise Sequence Alignment (https://www.ebi.ac.uk/jdispatcher/psa/emboss_needle). BLAST software (http://www.ncbi.nlm.nih.gov/blast/) compared the nucleotide sequences analyzed in the present study with sequences available in GenBank. This search revealed that the patient's isolate belonged to DTU TcI with a DNA sequence similar to that of the strain published in GenBank, access EU302215.1^22^. Electropherogram detected a single DTU in the patient's blood culture. However, the possibility of selecting a specific DTU for cultivation cannot be ruled out. Selective pressure from the in vitro environment may favor the clonal expansion of specific variants, thus reducing the genetic heterogeneity of the original isolate. However, this may require a considerable manipulation time^6^. The DNA sequence of COII of *T. cruzi* isolated from the patient, the BLAST results, and the COII sequences of standard strains from TcI to TcVI were used for alignment and construction of the phylogenetic tree using the software MEGA version 12^23^, as shown in Figure 1E.

## LITERATURE REVIEW

A total of 76 cases of CD in immunosuppressed patients, with or without clinical reactivation, have been reported, along with the identification of the corresponding *T. cruzi* DTUs^9^ ^-^ ^19^, of which 23 were unspecified (transplanted or coinfected with *T. cruzi*/HIV)^14^, 11 were transplanted, and 1 patient had lymphoma^17^. The remaining 41 patients were coinfected with *T. cruzi*/HIV (Table 1). Notably, eight coinfected patients were receiving antiretroviral therapy and did not present with reactivation^18^. Considering all patients who had their DTU determined and excluding genotyping results with ambiguous identification, such as those suggesting a TcII - TcVI, TcII/V/VI, or TcV/VI pattern, TcI, TcII, TcV, and TcVI were identified in pure and mixed infections of two DTUs in 53 patients. TcV was the most prevalent, found in 28 (52.8%) patients^11^ ^,^ ^13^ ^,^ ^14^ ^,^ ^17^ ^,^ ^19^, followed by TcI in 18 (34%) patients^12^ ^-^ ^15^ ^,^ ^17^, TcVI^9^ ^,^ ^11^ ^,^ ^14^ ^,^ ^19^ and TcII^11^ ^,^ ^14^ ^-^ ^18^ in 9 (17%) patients (Table 1). Pure infection by only one DTU was detected, in descending order, in 25% of patients infected with TcV, 21,1% of those infected with TcI, 19% of those infected with TcII, and 4.8% of those infected with TcVI, and mixed infections by two DTUs were detected in 15 (19.7%) patients. These DTUs were mainly detected in 51 (67.1%) patients from Argentina (TcV, TcVI, TcII, and TcI), 11 (14.5%) patients from Brazil (TcII and TcVI), 3 (4.0%) patients from Paraguay, 2 (2.6%) patients from Bolivia (TcI and TcV) and 1 (1.3%) patient from Colombia (TcI and TcII). In eight patients (10.5%), DTU was not determined (Table 1). TcV is the most common DTU in the Southern Cone of South America and is found in approximately 50% of the patients in Argentina^7^. Additionally, TcI, which is widely distributed and prevalent in the Andes, is the DTU most frequently associated with CNS involvement in patients with HIV, especially in severe reactivations^7^ ^,^ ^11^ ^,^ ^15^.

A survey of 48 HIV-positive patients with or without CNS involvement identified distinct clinical outcomes and the associated *T. cruzi* DTUs (Table 3). Among them, 26 (54.2%) survived, 11 (22.9%) died, and 11 (22.9%) had unknown outcomes^13^ ^,^ ^17^.

**TABLE 3:** Clinical outcomes, analysed samples, and *Trypanosoma cruzi* discrete typing units (DTUs) in human immunodeficiency virus-positive patients with or without central nervous system reactivation of Chagas disease (RCD).

Patients^a^	Sample tested	DTU^b^	Outcome	References
1 (27y M)^c^	Blood	TcVI^d^	Death	^9^
2 (63y M)	Blood and CSF^e^	TcII-VI^f^	Discharged into a coma	^10^
3 (29y M)	Blood and brain	TcV (blood), TcII/VI (brain)	Alive after 30 months post-RCD	^11^
4 (41y M)	Blood and CSF	TcI + TcV/VI (blood), TcI (CSF)	Death	^12^
5	Blood and CSF	TcII/V/VI	Encephalitis, treated with BZ^g^ and seroconverted	^13^
6-7^h^	Blood	TcV	CD^i^ indeterminate form	^13^
8-11^h^	Blood	ND^j^	CD indeterminate form	^13^
12 ^h^	Blood	TcV	Died 72 h post-delivery	^13^
13 ^h^	Blood	TcI + TcV	BZ-treated loss of follow-up	^13^
14-15^h^	Blood	TcV	Alive	^13^
16	Blood and brain biopsy	TcV (blood), TcII (brain)	Alive	^13^
17	Blood and CSF	TcI + TcV (blood), TcI (CSF)	Death	^13^
18	Blood and brain necropsy	TcV (brain)	Death	^13^
19^h^-20	Blood	TcV	Unknown	^13^
21	Blood and CSF	TcV	Unknown	^13^
22	CSF	TcII/V/VI	Unknown	^13^
23	Blood	ND	Unknown	^13^
24	Brain	TcII	Death	^14^
25 (34y F)^c^	Brain and heart	TcI (Brain), TcI + TcII (Heart)	Death	^15^
26^k^ (42y F)	Blood	TcII	Survived with mild sequelae (ART therapy)	^16^
27-32^k^	Blood and skin biopsy	TcI (3), TcII (1), TcV (1), TcII/VI (1)	Unknown	^17^
33-40^l^	Blood culture	TcII (2), TcII + TcV/VI (1)	Alive	^18^
41 (42y M)	Blood and CSF	TcVI	Died after 40 d de BZ, sequelae of brain injury	^19^
42 (65y M)	Blood and CSF	TcV + TcVI	Died after 15 d of BZ, concomitant CNS infections	^19^
43 (65y F)	Blood and CSF	TcV	Completed 60 d of BZ. Alive after 2 y post-RCD	^19^
44 (59y M)	Blood and CSF	TcV	Extended to 90 d of BZ. Recovered without neurological sequelae. Started ART. Alive 1 y post-RCD	^19^
45 (69y M)	Blood and CSF	TcV	Died after 15 d of BZ, and 25 d on mechanic ventilation	^19^
46 (39y F)	Blood and CSF	TcV + TcVI	Completed 30 d of BZ and 30 d of nifurtimox. Alive after 6 y post-RCD	^19^
47 (40y F)	Blood and CSF	TcV + TcVI	Completed 60 d of BZ. Alive after 6 y post-RCD	^19^
48 (50y M)	Blood and CSF	TcV + TcVI	Died after 21 d of BZ due to acute abdominal complication	^19^

a Coinfected mothers (5-11) and some of their infants (de 12 a 13); ^b^ genetic groups TcI, TcIIb, TcIIc, TcIIa, TcIId and TcIIe correspond to the current DTUs TcI, TcII, TcIII, TcIV, TcV and TcVI, respectively^20^; ^c^ age **y:** years and sex **M:** male; **F:** female; ^d^ previously classified as genetic group II (similar to the CL strain)^20^; ^e^ CSF = cerebrospinal fluid; ^f^ corresponding to the parasitic lineage 1 (*T. cruzi* II)^20^; ^g^ BZ = benznidazole; ^h^ Absent or unknown reactivation of Chagas disease; ^i^ **CD:** Chagas disease; ^j^ **ND:** not determined; ^k^ clinical reactivation in the context of immunosuppression; ^l^ under antiretroviral therapy (and one BZ-treated).

Among the 26 survivors, outcomes varied: one was discharged in a comatose state^10^, one remained alive 30 months postreactivation^11^, one patient with encephalitis, BZ-treated and seroconverted, six were classified as having the indeterminate form of CD, one was treated with loss of follow-up, and three were recorded only as “alive”^13^. Additionally, four patients completed BZ (or BZ + nifurtimox) treatment for 60-90 d^19^. Fifty percent (6/12) of all the BZ-treated patients survived or had negative diagnostic tests ^13^ ^,^ ^18^ ^,^ ^19^. Among those who died, four received BZ but did not complete the full course of treatment, succumbing to neurological or other complications^19^ (Table 3). Of the patients (20.8%) undergoing antiretroviral therapy (ART), all survived, and eight did not present with RCD, highlighting the effectiveness of ART in preventing reactivation^16^ ^,^ ^18^ ^,^ ^19^.

DTUs were unequivocally identified in 34 cases, of which 23 (67.7%) involved a single DTU and 11 (32, 4%) involved mixed infections. The most prevalent DTU was TcV, found in 21 (61.8%) patients, followed by TcII found in eight (23.5%), TcI in seven (21.9%), the combination TcV+TcVI in four (11.8%), and TcVI alone in two (5.9%) (Table 3). Most patients originated from Argentina and Paraguay, where TcV predominates, suggesting an association between patient origin and the geographic distribution of DTU^7^.

Among the 34 cases in which DTU or combinations of DTUs were identified, nine (26.5%) had no reported clinical outcomes. Among the patients with available outcome data, 11 (32.4%) died. All four DTUs were detected in fatal cases. However, case lethality rates varied according to DTU in the following decreasing order: TcI (100%; 3/3), TcVI (66.7%; 4/6), TcV (35.3%; 6/17), and TcII (28.6%, 2/7) (Table 3). Mixed infections were reported in all four cases in which DTU TcI was identified (cases 4, 13, 17, and 25). Nevertheless, in the three cases that progressed to death, TcI was the only DTU detected in the CSF/brain, a finding consistent with the present case. Although the higher lethality observed may be related to CNS involvement, the limited number of cases and the frequent occurrence of mixed infections highlight the need for further studies to confirm the potential associations with specific DTUs, particularly TcI.

Among the 26 surviving patients, DTU characterization was not available for 14 (53.9%), whereas it was unequivocally identified in 12 (46.2%). Among these, TcV was the most frequent, detected in nine patients (75%), followed by TcVI in two (16.7%), and TcII in one (8.3%) (Table 3). Additionally, a mixed infection with TcI and TcV was reported in a patient treated with BZ, who was subsequently lost^13^.

In this review, no clear association was observed between DTU and response to BZ treatment or between DTU and tissue tropism, as all four DTUs were detected across various sample types (blood, brain tissue, CSF, heart tissue, and skin), with TcV being particularly frequent (Table 3). However, TcV alone was detected in 14 blood samples and 6 brain/CSF samples, and TcI alone was detected in 4 brain/CSF samples.

**Ethical aspects:** This study was approved by the Research Ethics Committee / National Research Ethics Committee, Opinion Number: 5.989.993.

## DISCUSSION

Although RCD is recognized as an AIDS-defining condition^1^ ^,^ ^2^ the as far as our knowledge, this is the first report of Chagas meningoencephalitis diagnosed in a live HIV patient presenting with TcI infection in Brazil. However, we cannot rule out the possibility that the patient harbors other DTUs, in addition to TcI, since the blood culture can select a particular DTU with a greater capacity for growth in the culture medium to the detriment of others.

In the present case, the detection of DTU TcI was achieved via blood culture of the patient's blood while still alive, while in the Colombian patient, it was achieved in postmortem heart (TcI + TcII) and brain tissue (TcI) (Tables 1 and 3)^15^. Additionally, mixed infections of the four DTUs (TcI, TcII, TcV, and TcVI) have been found in different biological materials (blood, heart, brain, and CSF) and in approximately 15 patients from Argentina, Bolivia, Colombia, Brazil, and Paraguay^13^ ^-^ ^15^ ^,^ ^17^ ^,^ ^19^. All four DTUs were detected in the CSF/brain.

The direct association between *T. cruzi* DTU and the clinical manifestations of CD remains poorly understood. This case occurred in a Brazilian state, where TcII was predominant in patients with chronic CD^7^ ^,^ ^24^. Additionally, three other cases in Brazil identified pure infection with TcII in the blood of one patient with CNS reactivation^16^ and two patients without reactivation under antiretroviral therapy, in addition to mixed infection of TcII + TcV/VI in a fourth patient^18^ (Table 3), highlighting the importance of considering the prevalence of different *T. cruzi* DTUs in the region in cases of HIV coinfection. Notably, the absence of TcIII and TcIV in cases of RCD, corroborating the findings of Cura et al. (2012)^14^, which may have significant implications for understanding the epidemiology and pathogenesis of the disease in HIV coinfected patients.

Upon admission, the patient presented with hemiparesis, dysarthria, and gaze deviation, which initially led to a suspicion of stroke. However, the signs and symptoms observed during hospitalization were deemed more consistent with those of meningoencephalitis (decerebration, lowered level of consciousness, and hypertonia)^1^ ^-^ ^3^, and thus, an investigation into the cause was conducted.

CD is considered a neglected disease^2^ ^,^ ^3^; thus, the lack of resources and infrastructure in endemic areas can impede the diagnosis of affected individuals. The diagnosis of nonclassic cases such as RCD is even more challenging.

The exclusion of differential diagnoses is essential for all immunocompromised patients in whom CNS damage is suspected^2^ ^,^ ^19^. Infectious and ischemic processes cannot be differentiated based on hypoattenuating focal lesions and mass effects on CT images because these are nonspecific findings. In the present case, RCD was diagnosed after the detection of anti-*T. cruzi* antibodies in the serum and protozoa in the blood and CSF, as well as the exclusion of other causes. We could rule out the possibility that the CNS lesions were associated with HIV-related encephalitis; however, we could not rule out the possibility that they were related to neurotoxoplasmosis owing to the similarity in injury patterns between both infections^1^ ^,^ ^2^. Furthermore, the present case is similar to that reported by Hernández et al. (2014)^15^ in a Colombian patient. This is a typical example of RDC in an AIDS patient presenting with neurochagoma and coinfection with different DTUs. In this case from Colombia, TcI showed a specific tropism for the CNS, as observed in other studies^12^. Late diagnosis and confusion with cerebral toxoplasmosis contributed to the fatal outcome, and the authors advocated the formal inclusion of *T. cruzi* on the list of opportunistic pathogens for HIV management. Neurotoxoplasmosis is a common confounding factor in cases of RCD meningoencephalitis^15^, which can delay specific treatment and worsen the prognosis. In this case, treatment for neurotoxoplasmosis was initiated 5 d before the identification of *T. cruzi*.

In Brazil, serological testing of anti-*T. cruzi* antibodies be conducted in all cases of CNS damage where the patient is HIV-positive, which is little done^1^ ^,^ ^3^. A definitive diagnosis of RCD is only possible, however, with the analysis of CSF. The characteristics include a clear appearance, pleocytosis with slight lymphocytosis, normal or slightly reduced glycorrhachia, and a slight increase in proteins^3^. Of particular importance, as seen in the present case, is the visualization of *T. cruz*i trypomastigote forms using direct microscopy of body fluid samples (blood and CSF).

Although direct parasitological tests of the blood and CSF were negative during follow-up, this does not rule out therapeutic failure, as these conventional methods have low sensitivity and may not detect residual drug-resistant *T. cruzi* subpopulations. In the present case, parasite persistence was confirmed by the isolation and genotyping of viable forms in blood culture, suggesting BZ resistance. qPCR, a more sensitive tool for detecting low-level parasitemia and monitoring treatment response in immunosuppressed patients, might have identified this earlier^2^ ^,^ ^5^. However, the absence of this methodology in our laboratory is a limitation of the present study.

The relationship between parasitemia and CD4 + T lymphocyte count has already been investigated in *T. cruzi*/HIV coinfection^25^. A direct relationship between the level of parasitemia (quantified by PCR) and viral load and an inverse relationship between parasitemia and the level of CD4+ T lymphocytes, or the CD4+/CD8+ ratio, in coinfected individuals has already been demonstrated^1^ ^,^ ^4^ ^,^ ^5^. It is estimated that 20% of coinfected people will have RCD; however, when considering individuals with a CD4 count of <200 cells/mm³, this percentage increases to around 80%^1^ ^,^ ^4^. Consistent with this, in the present case, the CD4 count reached 39 cells/mm³ prior to treatment. 

In general, opportunistic infections are treated before initiating ART, thus avoiding the development of Immune Reconstitution Inflammatory Syndrome, especially if there is a past or current history of coinfections or opportunistic infections^1^. Treatment of RCD in HIV-positive patients must be initiated immediately; 5 mg/kg/day benznidazole in two doses, not exceeding 300 mg/day, for up to 60 d is recommended^1^ ^-^ ^3^. In the present case, treatment with benznidazole began 2 d after the diagnosis of CD (22 d.a.a.), as the detection of trypomastigotes in the CSF is considered the gold standard for chagasic meningoencephalitis^2^. After starting treatment, the parasitic forms were no longer detected in the CSF (Table 2). Despite this, the RCD treatment lasted 76 d, which was justified by the absence of clinical improvement.

The prognosis of patients with RCD and CNS involvement is unfavorable, with mortality rates after reactivation ranging 79-100%^1^. However, when treatment is initiated within 30 d, the survival rate can reach approximately 80%¹^,^³. Recent evidence has highlighted that early diagnosis, immediate antiparasitic treatment, regular patient follow-up, and epidemiological surveillance are essential for the management of *T*. *cruzi*/HIV coinfection and RCD. This is particularly critical in cases of meningoencephalitis, which is associated with high mortality, as all untreated patients have been reported to die shortly after diagnosis^25^. Although the patient was treated with benznidazole shortly after the diagnosis of RCD, he died. It is possible that infection with *T. cruzi* DTU TcI, reported to have a lower susceptibility to the drug^8^-may have contributed to the unfavorable clinical outcomes. In this case, the late diagnosis of coinfection, delay in initiating antitrypanosomal treatment, and ART likely affected the outcome. 

Mixed infection with TcII + TcV/VI was detected in one *T. cruzi*/HIV coinfected subject from São Paulo, Brazil, with a significant increase in CD4 T-cells counting and a decrease in viral load, which became undetectable over the years after ART^18^. Experimental studies have shown that TcII (clonal genotype 32) and TcVI (zymodeme B) are more drug-sensitive, TcV (clonal genotype 39) is partially drug-resistant, and TcI (mainly clonal genotype 20) is drug resistant^8^. The literature review did not demonstrate a clear association between DTU and the response to antiparasitic treatment, which may be explained by the fact that the efficacy of BZ relies on the host’s immune response. In the present case, this response was compromised, as indicated by a CD4 count below 100 cells/mm³ (Table 2). Therefore, in addition to early diagnosis and treatment, genotyping of *T. cruzi* DTU(s) present in patients before CD reactivation is highly recommended.

Accurately determining the likely route of infection, history of migration, or history of prior blood transfusion was not possible. However, the two municipalities where the patient lived were in Northwestern Paraná, a region with high climatic and landscape suitability for the CD vector^26^. Furthermore, this case occurred in a Brazilian state where TcI has rarely been isolated from patients with CD and TcII predominates in immunocompetent patients with chronic CD, although TcI also occurs in the sylvatic transmission cycle in pure and mixed infections with TcII in opossums and triatomines^24^. Therefore, this may be important in immunosuppressed patients and may be related to a worse prognosis. However, further studies are required to confirm this hypothesis.

This report describes the first documented case of Chagas meningoencephalitis in which the TcI DTU of *T. cruzi* was diagnosed in a living HIV-positive patient in Brazil, which resulted in death despite treatment with BZ. Although parasitemia was not monitored by qPCR during or after treatment, the diagnosis of RCD was established based on the clinical course and laboratory findings, including the detection of *T. cruzi* in both CSF and peripheral blood. Given the high clinical suspicion of reactivation, the patient was treated with BZ, following established protocols for such cases. Despite therapeutic intervention, the patient died approximately 1 month after hospital discharge in a clinical context consistent with reactivated Chagasic meningoencephalitis.

This fatal outcome reinforces the lethality of RCD with CNS involvement, particularly associated with TcI DTU, which is more frequent in neurological cases^11^ ^,^ ^13^ and is potentially less responsive to BZ^7^ ^,^ ^8^. Notably, serological testing for CD should be performed on all patients with neurological signs and symptoms, particularly in individuals with a compatible epidemiological history, such as those of rural origin and those who have lived in endemic areas in the past, such as Northwest of Paraná. The omission of CD from the differential diagnosis of opportunistic CNS infections and acute myocarditis frequently results in delayed initiation of specific anti-*T. cruzi* therapy. The clinical similarity with other opportunistic infections, such as neurotoxoplasmosis, lack of standardized protocols for parasite genotyping in Brazil, delayed diagnosis, and lack of specific intervention. This case highlights the urgent need for (1) systematic screening of *T. cruzi* in HIV+ patients with neurological symptoms, (2) implementation of quantitative PCR and genotyping of the CSF for early identification of high-risk DTUs (such as TcI), and (3) evaluation of alternative therapies for reactivation by potentially resistant strains. The underreporting of DTUs in Brazil requires expanded epidemiological surveillance, especially in endemic regions where parasite diversity can influence outcomes.

## Data Availability

Research data is only available upon request.
